# Supplementary material for: A drug design strategy based on molecular docking and molecular dynamics simulations applied to development of inhibitor against triple-negative breast cancer by Scutellarein derivatives
Source: PLoS One. 2023 Oct 12;18(10):e0283271. doi: 10.1371/journal.pone.0283271 (PMC10569544; doi:10.1371/journal.pone.0283271)
Supplement: S1 Table — (DOCX) [file pone.0283271.s001.docx]

Substitutes Tabe: S1

| Ligands Interaction with amino acid residues and their bond distance Triple-negative Breast cancer (PDB ID 7L1X) | | | | | |
| --- | --- | --- | --- | --- | --- |
| Ligand No | Hydrogen bond | | Hydrophobic bond | | Van der Waals bond |
|  | The interacting residue of amino acid | Distance, A֯ | The interacting residue of amino acid | Distance, A֯ |  |
| DM01 | A: VAL116  A: GLU81  A: VAL116  A:ASP175 | 2.19855  2.90948  2.18948  3.08924 | A: LEU45  A: ILE174  A: ILE174  A: MET163  A: MET163  A: PHE113  A: VAL66  A: VAL66  A: ILE174  A: LYS68  A: ILE95 | 3.82566  3.69856  3.99331  4.05365  4.11301  4.361  5.30488  4.09747  4.96992  5.13467  5.18446 | Absent |
| DM02 | A:LEU45:O | 2.37567 | A:LEU45  A:ILE174  A:MET163:  A:HIS115  A:VAL53  A:VAL66  A:ILE174  A:VAL53  - A:VAL66  A:LYS68  A:LEU45 | 3.82871  3.76917  4.06554  4.98572  5.04653  4.60832  5.43038  5.13792  5.47377  5.07494  5.21386 | Absent |
| DM03 | A:LEU45 | 2.44953 | A:LEU45  A:ILE174  A:MET163  A:HIS115  A:LEU45  A:VAL53  A:VAL66  A:VAL53  A:VAL66  A:LYS68  A:LEU45 | 3.81103  3.70586  4.13839  4.65361  5.29355  5.02144  4.58938  5.07691  5.35123  5.18086  5.37578 | Absent |
| DM04 | A:TYR50  A:HIS160 | 2.27175  3.716 | A:ASP175  A:LEU45  A:GLY48  A:VAL66  A:VAL53  A:ILE95  A:ILE174 | 3.55012  3.68277  3.67446  3.68683  4.82273  5.26873  4.1437 | Absent |
| DM05 | A:ARG43  A:ARG43  A:LEU45  N:UNK0 | 2.94033  2.90644  2.40961  2.8833 | A:LEU45  A:ILE174  A:MET163  A:HIS115  A:VAL53  A:VAL66  A:ILE174  A:LEU45  A:VAL53  A:VAL66  A:LYS68 | 3.85935  3.77229  4.05546  5.03473  5.04645  4.63137  5.42747  5.11571  5.14133  5.4629  5.0798 | Absent |
| DM06 | A:ASP120  A:LEU45 | 2.71038  2.877 | A:LEU45  A:ILE174  A:MET163  A:PHE113  A:HIS115  A:LEU45  A:VAL53  A: VAL66  A: LEU45  A: VAL53  A: VAL66  A: LYS68 | 3.82944  3.72841  4.10071  4.98929  4.5891  5.32308  5.04731  4.62024  5.3522  5.00522  5.36903  5.11872 | Absent |
| DM07 | A:ASN117  A:SER51  A:ASP175  A:GLU81 | 2.38493  3.69923  3.71649  3.61764 | A:VAL53  A:ILE174  A:MET163  A:PHE113  A:HIS160  A:HIS115  A:LYS68  A:ILE95  A:PHE113  A:TRP176  A:LEU45  A:LEU45  A:VAL53  A:MET163  A:VAL66  A:LYS68  A:ILE95 | 3.86786  3.97073  3.92427  4.48022  4.70931  4.57882  5.25261  5.3385  3.91933  5.36881  4.93745  4.37779  5.29924  5.37743  5.05834  5.39572  5.15724 |  |
| DM08 | A:ARG43  A:LYS68  A:PHE113 | 2.35906  2.52408  3.12328 | A:LEU45  A:ILE174  A:MET163:  A:PHE113  A:HIS115  A:LEU45  A:VAL53  A:VAL66  A:LEU45  A:VAL53  A:VAL66  A:LYS68 | 3.78672  3.70656  4.17296  5.045  4.45932  5.19606  4.96931  4.66006  5.35794  4.95738  5.33408  5.14437 |  |
| DM09 | A:LYS68  A:LYS68  A:ASN117  A:ASN118  A:ASN118  A:ASN117 | 2.5669  2.99089  2.56571  2.55087  1.88894  3.49351 | A:LEU45  A:ILE174  A:MET163  A:HIS115  A:LEU45  A:VAL53  A:VAL66  A:LEU45  A:VAL53  A:VAL66  A:LYS68 | 3.91065  3.70804  4.23547  4.65303  5.1627  4.85275  4.73883  5.36059  4.94898  5.36764  5.11945 |  |
| DM10 | A:ARG43  A:LYS68  A:LYS68  A:ASN117  A:ASP120  A:THR119 | 2.26295  2.47657  2.89742  2.17253  2.02882  3.11221 | A:ILE174  A:MET163  A:HIS115  A:HIS160  A:LEU45  A:LEU45  A:VAL53  A:VAL66  A:LEU45  A:VAL53  A:VAL66  A:LYS68 | 3.74385  4.16434  4.57778  4.97585  5.25646  4.41441  4.89593  4.77848  5.33975  4.94175  5.3771  5.0756 |  |
| Capecitabine | A:VAL116  A:ASN118  A:VAL116 | 2.34784  2.4012  2.66935 | A:LYS68  A:ILE174  A:PHE113 | 4.66953  4.20636  4.58008 | Absent |

| Table 4: Ligands Interaction with amino acid residues and their bond distance Triple-negative Breast cancer (PDB ID 5HA9) | | | | | |
| --- | --- | --- | --- | --- | --- |
| Ligand No | Hydrogen bond | | Hydrophobic bond | | Van der Waals bond |
|  | The interacting residue of amino acid | Distance, A֯ | The interacting residue of amino acid | Distance, A֯ |  |
| DM01 | A:SER203  A:ARG217 | 2.29875  2.00084 | A:GLU102  A:ASP105  A:TYR235  A:HIS201  A:HIS201 | 3.96055  4.97404  3.98982  4.8159  5.02267 | Absent |
| DM02 | A:SER322  A:GLN98  A:GLN98  A:TYR246 | 2.84528  2.69378  2.58541  3.77068 | A:GLU102  A:LEU323  A:TYR235  A:MET229 | 4.22515  3.68035  3.79321  4.9045 | Absent |
| DM03 | A:ASN73  A:HIS81  A:SER72 | 3.23146  2.17717  2.45356 | A:LEU90  A:LEU90  A:TYR76  A:PRO88  A:PRO88  A:PRO88 | 3.87553  3.94616  5.47845  3.8948  5.01151  5.33987 | Absent |
| DM04 | A:TYR246  A:TYR246 | 3.61711  3.64319 | A:GLU102  A:MET229  A:TYR235  A:TYR235  A:TYR246  A:ALA99 | 4.14285  3.59081  4.07119  5.61128  5.26332  5.2752 | Absent |
| DM05 | A:TYR246  A:TYR246 | 3.70172  3.51016 | A:GLU102  A:TYR235  A:TYR246  A:TYR246  A:LEU324  A:LEU323 | 4.09343  3.98535  5.91001  5.39953  4.55355  4.99009 | Absent |
| DM06 | A:SER72  A:TYR76 | 1.95905  3.02142 | A:LEU90  A:TYR76  A:LYS87  A:LEU90  A:PRO88  A:PRO88  A:LYS87  A:LEU69  A:PRO88 | 3.86845  5.51364  4.48946  4.33716  5.01516  3.92492  5.30683  5.24384  5.42386 | Absent |
| DM07 | A:TYR246  A:TYR246 | 3.56367  3.72456 | A:GLU102  A:GLU102  A:MET229  A:TYR235  A:TYR235  A:TYR246  A:MET229  A:TYR28  A:ALA99 | 4.24699  4.00753  3.54896  4.08376  5.45806  5.26487  4.95813  5.14718  5.29401 |  |
| DM08 | A:SER243  A:PHE236  A:GLY202  A:TYR246  A:SER203  A:SER203 | 3.09071  2.09376  2.2675  2.35382  2.34958  4.19114 | A:HIS201  A:TYR246  A:ARG217  A:ASP105 | 4.5269  3.88644  5.31766  3.54574 |  |
| DM09 | A:THR163  A:THR163  A:THR163  A:HIS161  A:HIS161 | 3.04896  2.99279  2.67932  2.24038  3.00484 | A:GLU171  A:HIS161  A:LYS158 A:LYS158  A:ARG154  A:VAL157  A:LYS158 | 3.82172  3.93199  4.90878  4.55876  5.22647  5.47865  5.06414 |  |
| DM10 | A:GLN98  - A:GLU102  - A:ASN245  - A:ASN245  A:ALA99  A:LYS242  A:ASN245  A:THR249 | 2.47834  2.65217  2.24521  2.07739  3.46859  3.64561  3.64933  3.25076 | A:GLU102  A:TYR28  A:HIS248  A:ALA99 | 3.79436  4.92535  5.00722  5.40151 |  |
| Capecitabine | A:ARG217  A:GLU102 | 2.89225  2.53439 | A:GLU102  A:TYR246  A:ILE211  A:HIS201  A:TYR235 A:TYR246 | 3.26964  3.61554  4.76438  5.3232  4.81214  3.69267 | Absent |

| [Note: TRP = Tryptophan, ASP = Aspartic acid, GLU = Glutamic acid, LEU = Leucine, THR = Threonine, ASN = Asparagine, GLN = Glutamine, PHE = Phenylalanine, ILE = Isoleucine, ARG = Arginine, VAL = Valine, SER = Serine, PRO = Proline, GLY = Glycine, HIS = Histidine, LYS = Lysine, TRP = Tryptophan, CYS = Cysteine, MET = Methionine.] |
| --- |
